# Morning spot urinary cortisol-to-creatinine ratio: a novel screening tool for assessing excess cortisol secretion

**DOI:** 10.3389/fendo.2025.1663619

**Published:** 2025-11-19

**Authors:** Yingning Liu, Ping Zhang, Simin Zhang, Ying Gao, Yiqing Mu, Hong Lian, Qian Ren, Xiaoling Cai, Xianghai Zhou, Xueyao Han, Linong Ji, Xiantong Zou

**Affiliations:** 1 Department of Endocrinology and Metabolism, Peking University People’s Hospital, Peking University Diabetes Center, Beijing, China; 2 Department of Medical Information Center, Peking University People’s Hospital, Beijing, China

**Keywords:** urinary cortisol-to-creatinine ratio, dexamethasone suppression test, hypercortisolism, diabetes mellitus, screening tool

## Abstract

**Context:**

Current screening methods for hypercortisolism face limitations in clinical practice.

**Objective:**

Our study proposes and validates a novel biomarker, the morning spot urinary cortisol-to-creatinine ratio (UCCR), as a simpler alternative for assessing excess cortisol secretion.

**Methods:**

This cross-sectional study was conducted in Chinese hospitalized patients, comprising a cohort of 167 patients who underwent the 1mg overnight dexamethasone suppression test (1mg DST). Urinary free cortisol level (UFC) and creatinine were measured using morning spot urine, and UCCR was subsequently calculated. Receiver operating characteristic (ROC) curve analysis was used to assess the performance of these parameters in predicting the results of the 1mg DST.

**Results:**

Morning spot UCCR showed significant correlations with 24-hour UFC and was independently associated with a positive 1mg DST result. The ROC AUCs for morning spot UCCR were 0.642 (0.549-0.734) and 0.762 (0.665-0.859) in predicting cortisol >1.8 µg/dL and >5.0 µg/dL post-1mg DST, respectively, comparable to those of 24-hour UFC and UCCR. Morning spot UCCR demonstrated high sensitivity of 71.4% and 86.4% for predicting post-DST cortisol >1.8 µg/dL and 5.0 µg/dL, respectively. The negative predictive value (NPV) of morning spot UCCR was 83.5% for cortisol >1.8 µg/dL and 96.8% for >5.0 µg/dL post-1mg DST. A significant reduction in ROC AUC was observed in males, with a borderline decrease noted in patients with diabetes.

**Conclusions:**

Morning spot UCCR is a reliable alternative for the initial evaluation of cortisol secretion and is particularly useful for excluding cortisol excess. Nonetheless, caution is advised when applying this test in males or patients with diabetes.

## 1 Introduction

Glucocorticoids are steroid hormones involved in multiple physiological processes and are known to impair glucose metabolism and glycemic control (1, 2). Overt Cushing syndrome encompasses a broad spectrum of clinical manifestations resulting from prolonged cortisol excess. Typical features include central obesity, facial rounding, supraclavicular and dorsocervical fat pads, skin fragility with easy bruising and wide purple striae, proximal muscle weakness, hypertension, glucose intolerance or diabetes, and neuropsychiatric symptoms, among others. However, mild autonomous cortisol secretion—defined as hypothalamic-pituitary-adrenal (HPA) axis dysregulation without overt signs of cortisol excess (3)—has only recently been recognized for its impact on elevated cardiovascular events and mortality (4, 5). A recent meta-analysis reported a 11.7% prevalence of autonomous cortisol secretion among subjects with adrenal incidentalomas (6). In patients with primary aldosteronism, the prevalence of autonomous cortisol secretion was 21.9% in a cohort study and systematic review (7). Furthermore, multiple studies have shown that mild autonomous cortisol secretion is significantly more prevalent in patients with diabetes than in the general population (8–10). The Hypercortisolism in Patients with Difficult to Control Type 2 Diabetes Despite Receiving Standard-of-Care Therapies: Prevalence and Treatment with Korlym (CATALYST) trial recently reported a 24% prevalence of endogenous hypercortisolism among patients with poorly controlled type 2 diabetes, much higher than previously expected (11). Therefore, early and accurate screening for hypercortisolism is warranted in patients with difficult-to-control diabetes, particularly when additional signs of cortisol excess are present.

Current guidelines for Cushing’s syndrome recommend 24-hour urinary-free cortisol (UFC), late-night salivary cortisol (LNSC), and the dexamethasone suppression test (DST) as the first-line screening methods (12, 13). The 1mg overnight DST is strongly recommended for screening mild autonomous cortisol secretion in patients with adrenal incidentalomas. In the updated guideline, patients without clinical signs of overt Cushing’s syndrome but with serum cortisol levels >50 nmol/L post dexamethasone were classified as having “mild autonomous cortisol secretion” (14). However, current screening methods face limitations in clinical implementation, operational challenges, and potential interference. Nowadays, LNSC testing has not been widely available in some regions. The accuracy of 24-hour UFC depends on the proper sample collection by patients, posing challenges for its use in outpatient settings. DST, the most sensitive screening method employed in clinical practice (15, 16), requires patients to take dexamethasone and undergo blood sampling at precise times, which demands good adherence. Additionally, dexamethasone administration during the DST may transiently affect glucose management and complicate medication adjustment in hospitalized patients with pre-existing difficulties in glycemic control (17, 18). Therefore, there is a pressing clinical need for a simple, non-invasive, and patient-friendly alternative for routine screening of hypercortisolism.

Urine creatinine concentrations are widely used to adjust or correct for urinary concentrations of chemicals or their metabolites (19). The rationale for measuring creatinine in a timed urine sample lies in its theoretically stable production from creatine in skeletal muscle and its predominant elimination via the kidneys. For example, the albumin-to-creatinine ratio in spot urine is widely accepted as a substitute for 24-hour albumin excretion and has been adopted in clinical guidelines (20, 21). By this means, this study introduces the morning spot urinary cortisol-to-creatinine ratio (UCCR) as a novel biomarker and evaluates its potential as a simplified alternative for evaluating daily cortisol secretion.

## 2 Materials and methods

### 2.1 Study population

**Figure 1:**
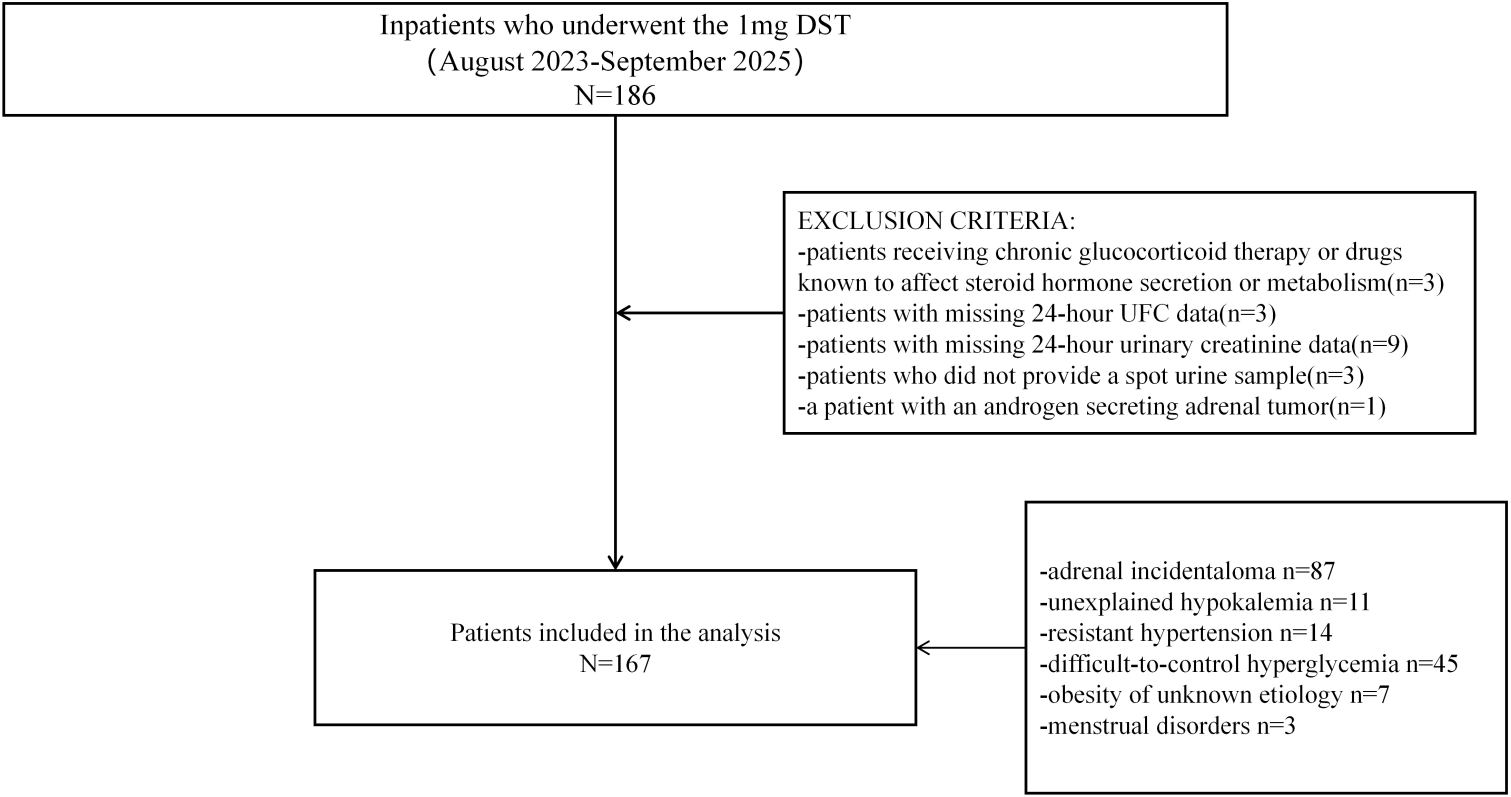
Study flow chart of participant inclusion and exclusion. A total of 186 hospitalized patients were initially recruited. Of these, 19 patients met the exclusion criteria and were excluded from the study. In total, 167 patients who underwent the 1mg DST were included in the analysis.

This cross-sectional, single-center study was conducted at the Department of Endocrinology and Metabolism of our hospital. Individuals admitted to the inpatient ward of the Department of Endocrinology and Metabolism between August 2023 and September 2025 who underwent the 1mg overnight dexamethasone suppression test (1mg DST) were initially recruited for the study. Indications for 1mg DST included incidentally discovered adrenal masses, clinical features suggestive of Cushing’s syndrome (e.g., centripetal obesity, wide purple striae), suspected primary aldosteronism, resistant hypertension, difficult-to-control hyperglycemia, and unexplained hypokalemia. We excluded patients receiving chronic glucocorticoid treatment or drugs known to affect steroid hormone secretion or metabolism. Additional exclusion criteria included patients with missing 24-hour urinary creatinine or 24-hour UFC data, as well as those who did not provide a spot urine sample. A patient with an androgen secreting adrenal tumor was also excluded to avoid potential analytical interference. In total, 167 patients were included in the cohort (**Figure 1**).

This study was conducted in compliance with the Declaration of Helsinki and was approved by the Ethics Committee of our hospital. The need for signed informed consent was waived.

### 2.2 Data collection and handling

With approval from the medical information center, data from eligible participants were extracted from the Clinical Data Application Platform, which contains medical information from over 13 million individuals who have attended our hospital. The data were processed and structured using Python 3.9.12, followed by manual verification.

Anthropometric measurements, including height, weight, neck, waist, and hip circumference, as well as blood pressure, were retrieved from the original medical records alongside sociodemographic characteristics, disease history, and medication history. Body mass index (BMI) was computed as weight in kilograms divided by height in meters squared. Additionally, laboratory test data were collected, including fasting plasma glucose (FPG), lipid profiles, renal function parameters, serum sodium, and serum potassium, which were measured using an automated biochemical analyzer. Hemoglobin A1c (HbA1c) levels were quantified using automated high-performance liquid chromatography, following a standardized procedure (Premier Hb9210, USA). Postprandial blood samples were also collected 2 hours after breakfast to measure C-peptide and glucose concentrations. Serum C-peptide levels were measured by the chemiluminescence method (Roche E411; Roche Diagnostics; Switzerland).

Both morning spot urine samples and 24-hour urine samples were collected. The 24-hour urine sample was collected by discarding the first morning void and subsequently collecting all urine produced over the following 24 hours. Spot urine samples were collected in the morning as the first midstream urine of the day. Creatinine concentrations in 24-hour urine samples were determined using an enzymatic assay, while those in spot urine samples were measured using the creatinine Jaffé method. Urine free cortisol levels were all quantified using a homogeneous enzyme immunoassay (Evermed, China), with calibrators traceable to Standard Reference Material (SRM) 921a from the National Institute of Standards and Technology (NIST), USA. The analytical measurement range was 20.0-1620.0ng/mL (r ≥0.990). According to the manufacturer’s documentation, excessively high concentrations of bilirubin, hemoglobin, or albumin may cause analytical interference or cross-reactivity. The inter-assay and intra-assay coefficients of variation (CV) of the cortisol metabolites measurements are<10.0%. The 24-hour urinary cortisol-to-creatinine ratio(ug/mmol, 24-hour UCCR) and spot urinary cortisol-to-creatinine ratio(ug/g, spot UCCR) were subsequently calculated. Participants also adhered to a standardized protocol for the 1mg overnight DST, with a baseline blood sample collected at 8:00 AM on Day 1. At midnight (0:00 AM) on Day 2, a 1mg oral dose of dexamethasone was administered, followed by the collection of a second blood sample at 8:00 AM to assess serum cortisol level. Serum cortisol concentrations were determined using a chemiluminescent immunoassay (Beckman Coulter, Inc., USA). The analytical measurement range was 0.4-60.0 ug/dL. Potential cross-reactivity may occur with structurally related compounds such as 11-deoxycortisol and other cortisol analogs. The intra-assay coefficient of variation (CV) was <6.7%, and the total imprecision was <7.9%. Both the standard cortisol post-1mg DST cutoff of 138 nmol/L (5.0 µg/dL) and the lower threshold of 50 nmol/L (1.8 µg/dL) were applied. The collection of 24-hour and spot urine samples was performed prior to dexamethasone administration.

### 2.3 Definitions

Diabetes was defined as a prior diagnosis of diabetes mellitus or a new diagnosis according to the Chinese Diabetes Society guidelines (22). Dyslipidemia in diabetes patients was defined based on the Chinese Diabetes Society guidelines as total cholesterol ≥4.50 mmol/L, triglycerides ≥1.70 mmol/L, low-density lipoprotein cholesterol (LDL-C) ≥2.60 mmol/L, high-density lipoprotein cholesterol (HDL-C) <1.30 mmol/L for women or <1.00 mmol/L for men, or ongoing antihyperlipidemic therapy (22). Dyslipidemia in patients without diabetes was defined according to the 2016 Chinese guideline for the management of dyslipidemia in adults (23). The diagnostic thresholds were ≥ 2.30 mmol/L for triglycerides, ≥ 6.20 mmol/L for total cholesterol or ≥ 4.10 mmol/L for LDL cholesterol, and <1.00 mmol/L for HDL cholesterol. Hypertension was defined as a systolic blood pressure ≥140 mmHg and/or a diastolic blood pressure ≥90 mmHg, in addition to existing hypertension treatment (24).

Overt Cushing’s syndrome was clinical diagnosed by experienced endocrinologists according to Chinese guidelines and confirmed through comprehensive evaluations, including biochemical assessments (serum cortisol circadian rhythm, low- and high-dose dexamethasone suppression tests, etc.), imaging (nuclear magnetic, adrenal imaging, etc.), and surgical biopsy when necessary (25).

### 2.4 Statistical analysis

Demographics and clinical characteristics of the cohort were expressed as mean ± standard deviation for continuous data with normal distribution, median (25th, 75th percentile) for skewed continuous variables, and number (percentage) for categorical variables.

Linear regression models were employed to evaluate the associations between morning spot UCCR, 24-hour UCCR, and 24-hour UFC. Morning spot UCCR and 24-hour UCCR were treated as independent variables, while 24-hour UFC was designated as the dependent variable in the regression models.

Logistic regression models were used to analyze the association between urinary cortisol tests and 1mg DST results. To identify key variables associated with the 1mg DST results, least absolute shrinkage and selection operator(LASSO) regression was performed. The most relevant variables were selected using a ten-fold cross-validation approach, which determined the optimal penalty term by minimizing prediction error(**Supplementary Figure 1**). Subsequently, the variables selected by LASSO regression and clinically relevant variables(sex, age and BMI) were used to adjust the logistic regression models.

The diagnostic accuracy of urinary cortisol tests in predicting cortisol levels post-1mg DST was assessed using receiver operating characteristic (ROC) curve analysis. Differences in area under the curve(AUC) values were compared using the DeLong test. Bonferroni’s correction was applied to control for a family-wise error rate in multiple comparisons. The optimal cut-off values were determined based on the Youden index, which was calculated as (maximum sensitivity + specificity) -1. Positive predictive value (PPV) is the proportion of true-positive results among all positive results, calculated as true positives/(true positives + false positives). Negative predictive value (NPV) is the proportion of true-negative results among all negative results, calculated as true negatives/(true negatives + false negatives).

To assess the potential impact of demographic and clinical factors on the diagnostic performance of urinary cortisol tests, stratified analyses were performed according to diabetes status, sex, age, and renal function. The definition of diabetes has been described previously. Sex was categorized as male or female according to medical record. Age was stratified into two categories: <60 years and ≥60 years, representing the younger and older subgroups, respectively. Renal function was stratified according to the estimated glomerular filtration rate (eGFR) calculated using the CKD-EPI equation, with eGFR values <90 mL/min/1.73 m²indicating reduced renal function and those ≥90 mL/min/1.73 m² indicating normal renal function.

All statistical analyses were performed with Python(Version 3.9.12), figures were generated using GraphPad Prism (Version 9.0.0), and the difference in ROCAUC was compared using MedCalc (Version 23.1.7).

## 3 Results

### 3.1 Association between 24-hour UFC and morning spot UCCR or 24-hour UCCR

Baseline characteristics of the cohort are summarized in **Table 1**. Among the 167 subjects in the cohort, 87 (52.1%) were identified with adrenal incidentalomas, 11 (6.5%) were eventually diagnosed with overt Cushing’s syndrome, and 26 (15.6%) were diagnosed with primary aldosteronism.

**Table 1:** Baseline characteristics of the cohort.

Parameter	Cohort (n=167)
Demographics	
Age, years	54.28 ± 14.04
Sex,n	male, 79 (47.3%)
Body mass index, kg/m^2^	27.6 ± 5.8
Systolic blood pressure, mmHg	142.7 ± 19.6
Diastolic blood pressure, mmHg	80.7 ± 13.0
Fasting plasma glucose, mmol/L	7.13 ± 2.84
HbA1c, %	7.72 ± 1.76
Serum potassium, mmol/L	3.86 ± 0.43
eGFR, ml/min	98.9 ± 20.9
Serum creatinine, umol/L	67.2 ± 26.2
Comorbidities	
Diabetes, n	103(61.7%)
Hypertension, n	122(73.1%)
Dyslipidemia, n	87(52.1%)
Obesity(BMI≥28), n	64(38.3%)
Adrenal incidentalomas, n	87(52.1%)
Mild autonomous cortisol secretion (MACS)	17(10.2%)
Non-functioning cortical adenomas	48(28.7%)
Aldosterone-producing adenoma (APA)	19(11.4%)
Pheochromocytoma	2(1.2%)
Myelolipoma	1(0.6%)
Overt Cushing syndrome, n	11(6.5%)
Primary aldosteronism, n	26(15.6%)
Laboratory measurements	
DST, µg/dL	1.19(0.81, 1.97)
DST<1.8µg/dL, n	118(70.7%)
1.8µg/dL<DST<5.0µg/dL, n	27(16.2%)
5.0µg/dL<DST, n	22(13.2%)
Urinary free cortisol, µg/24h	236.9(173.8, 342.0)
24-hour UCCR, ug/mmol	23.1(16.5, 31.1)
UCCR,ug/g	143.9(85.7, 231.6)

Data were expressed as means ± SD or median (interquartile range) for continuous variables with normal distribution or skewed distribution, respectively. Categorical variables were presented as number (percentage).

HbA1c, glycosylated hemoglobin, type A1C; eGFR, estimated glomerular filtration rate; DST, dexamethasone suppression test; UCCR, urinary cortisol-to-creatinine ratio.

**Figure 2:**
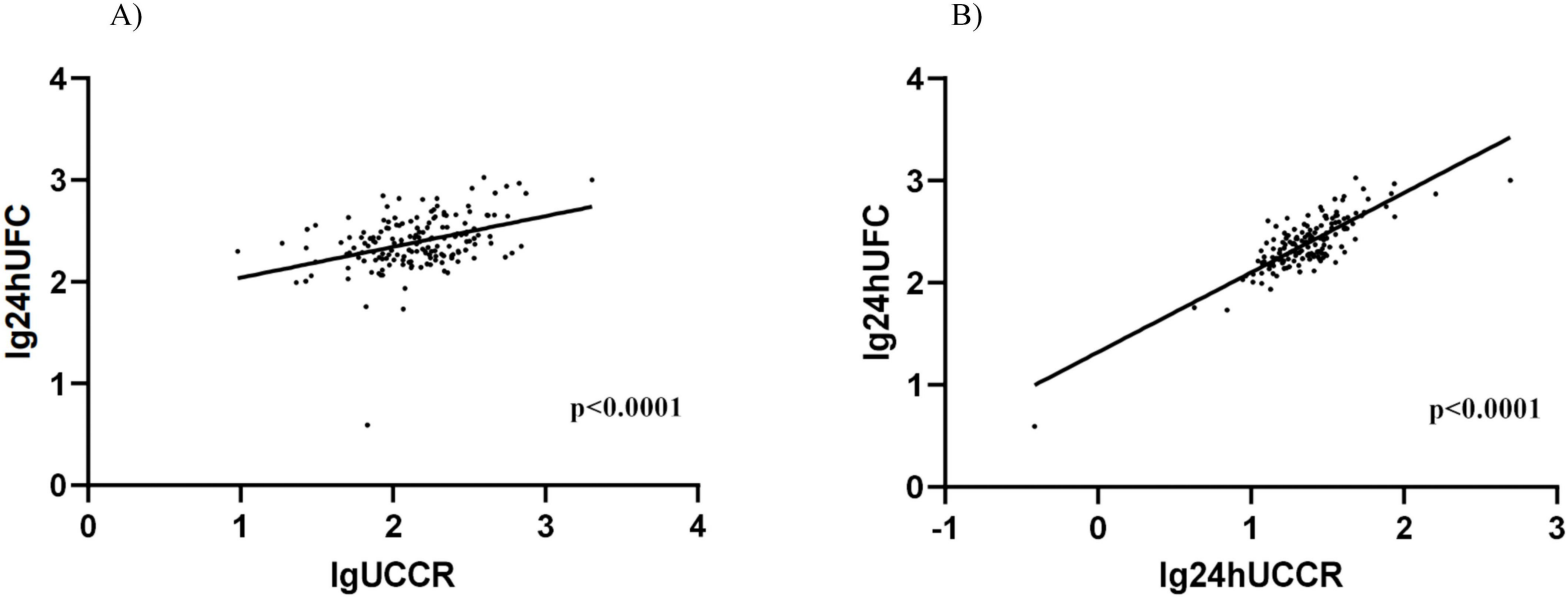
Association between morning spot UCCR and 24-hour UFC or 24-hour UCCR. Scatter plot depicting the relationship between morning spot UCCR and 24-hour UFC levels **(A)**, 24-hour UCCR and 24-hour UFC levels **(B).** Data from all participants (n=167) are shown, with log-transformed values analyzed by linear regression. DST-dexamethasone suppression test; UCCR-urinary cortisol-to-creatinine ratio; UFC-urinary free cortisol.

All participants underwent 24-hour UFC, 24-hour UCCR, and morning spot UCCR tests, so we performed a linear association analysis. Both morning spot UCCR (B = 0.303, P < 0.0001; **Figure 2A**) and 24-hour UCCR (B = 0.780, P < 0.0001; **Figure 2B**) exhibited a significant correlation with 24-hour UFC.

### 3.2 Association between urinary cortisol tests and 1mg DST results

LASSO regression identified 7 key variables predicting the 1mg DST result in the cohort: total cholesterol, diabetes status, sex, estimated GFR, serum sodium, 2-hour C-peptide, and HDL cholesterol (**Supplementary Table 1**). Logistic regression was then applied to evaluate the association between various urinary cortisol tests and 1mg DST results, as summarized in **Table 2**. Notably, all urinary cortisol tests, including morning spot UCCR, showed significant associations with an increased risk of positive 1mg DST results. At the threshold of >1.8 µg/dL, the adjusted odds ratios (ORs) were 1.56 (95% CI: 1.02-2.39) for morning spot UCCR, 2.17 (95% CI: 1.35-3.51) for 24-hour UFC, and 2.05 (95% CI: 1.27-3.31) for 24-hour UCCR. At the higher threshold of 5.0 µg/dL, the ORs increased substantially to 5.05 (95% CI: 2.19-11.66), 7.41 (95% CI: 3.01-18.25), and 7.02 (95% CI: 2.60-18.96), respectively. These results indicate an increased odds of having a positive 1 mg DST at increasing urinary cortisol levels.

**Table 2:** Binary logistic regression analysis for the association between urinary cortisol measurements and 1mg DST results.

Indicators	OR (95% CI) in crude	P value	OR (95% CI) adjusted	P value
Cortisol post 1mg DST >1.8 µg/dL				
24-hour UFC	2.268(1.458, 3.530)	<0.001	2.172(1.345, 3.505)	0.002
spot UCCR	1.740(1.190, 2.544)	0.004	1.562 (1.023, 2.385)	0.014
24-hour UCCR	2.058(1.295, 3.270)	0.002	2.051(1.271, 3.310)	0.003
Cortisol post 1mg DST >5.0 µg/dL				
24-hour UFC	6.867(3.229, 14.604)	<0.001	7.412(3.011, 18.247)	<0.001
spot UCCR	3.387(1.833, 6.258)	<0.001	5.050(2.188, 11.656)	<0.001
24-hour UCCR	5.524(2.495, 12.231)	<0.001	7.016(2.596, 18.961)	<0.001

Presented are results from logistic regression models with outcomes of cortisol post 1mg DST 1.8 µg/dL and>5.0 µg/dL, respectively. The urinary cortisol measurements, after log transformation and standardization, were used as independent variables. Model was adjusted with age, sex, BMI and counfounders identified by LASSO regression including total cholesterol, diabetes status, sodium, C-peptide at 120 minutes, and HDL cholesterol, estimated GFR.

DST, dexamethasone suppression test; UCCR, urinary cortisol-to-creatinine ratio; UFC, urinary free cortisol.

### 3.3 Diagnostic performance of urinary cortisol tests in predicting 1mg DST results

**Figure 3:**
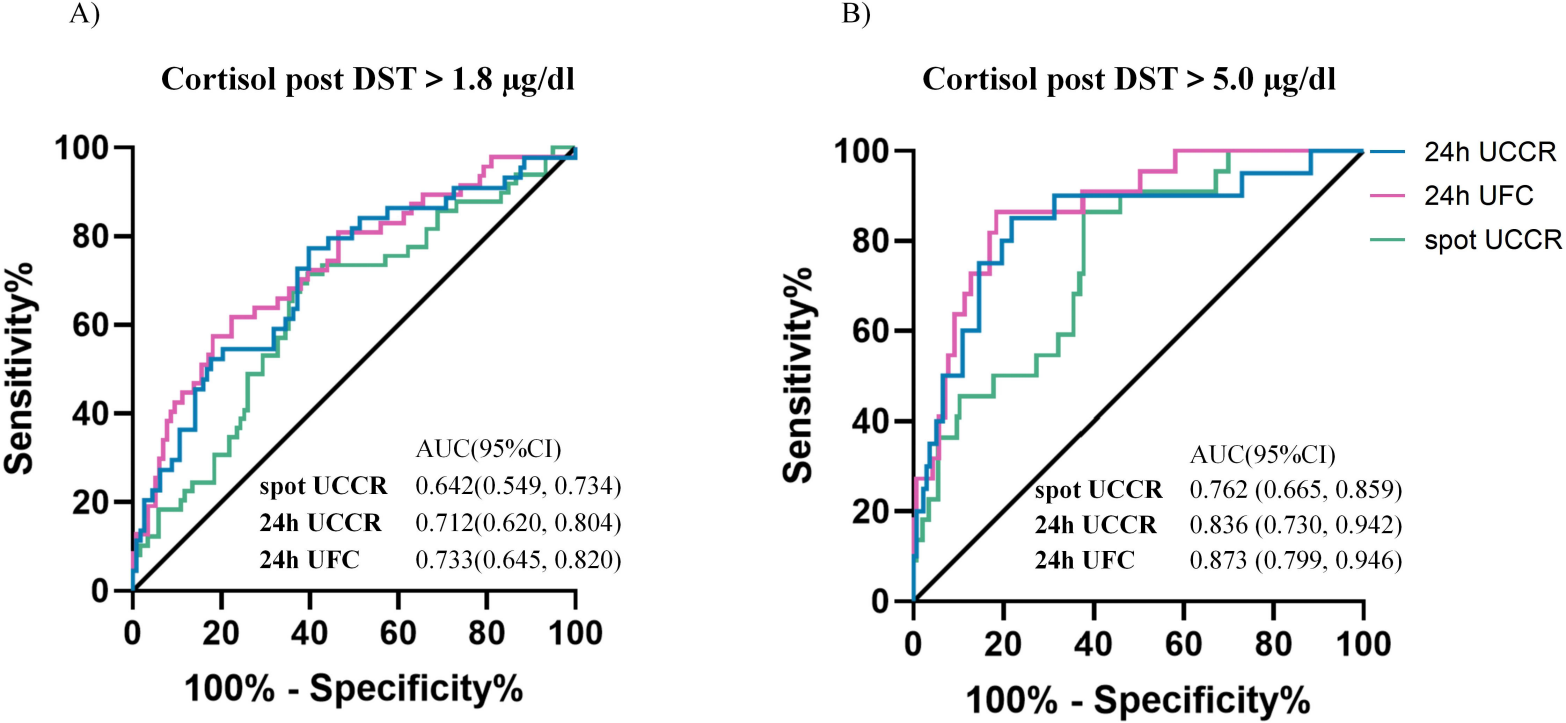
Diagnostic performance of urinary cortisol tests. ROC curves of morning spot UCCR, 24-hour UCCR and 24-hour UFC for predicting cortisol post 1mg DST >1.8 µg/dL **(A)** and >5 µg/dL **(B)**. Area under the curve (AUC) with exact binomial 95% confidence intervals (95% CI) were calculated. Solid lines represent the reference (AUC = 0.5, no discrimination). DST-dexamethasone suppression test; UCCR-urinary cortisol-to-creatinine ratio; UFC-urinary free cortisol.

Overall, morning spot UCCR demonstrated comparable discriminative ability to 24-hour UCCR and 24-hour UFC in predicting cortisol levels >1.8 µg/dL or >5.0 µg/dL post-1 mg DST, with AUCs of 0.642 (95%CI: 0.549-0.734) and 0.762 (95%CI: 0.665-0.859), respectively (**Figure 3**). The DeLong test revealed no significant differences in the pairwise comparisons of the ROC AUC values among the three cortisol parameters after Bonferroni correction (all p>0.05).

For cortisol >1.8 µg/dL post-1mg DST, 24-hour UCCR had the highest sensitivity and NPV. Morning spot UCCR, with a sensitivity of 71.4% and specificity of 60.2%, had lower specificity than 24-hour UFC but higher sensitivity. For predicting cortisol >5.0 µg/dL post-1mg DST, morning spot UCCR exhibited a sensitivity of 86.4% and specificity of 62.1%. The NPVs of morning spot UCCR for ruling out cortisol >1.8 µg/dL and >5.0 µg/dL post-1mg DST were 83.5% and 96.8%, respectively, similar to those of 24-hour UFC and 24-hour UCCR (**Table 3**).

**Table 3:** Diagnostic accuracy of morning spot UCCR, 24-hour UCCR and 24-hour UFC at various 1mg DST cutoff values.

Indicators	Cut-off value	Sensitivity (95% CI) (%)	Specificity (95% CI) (%)	PPV (95% CI) (%)	NPV (95% CI) (%)
Predicting cortisol post 1mg DST >1.8 µg/dL					
Spot UCCR, ug/g	>149.04	71.4 (56.7-83.4)	60.2 (50.7-69.1)	42.7 (35.9-49.7)	83.5 (76.1-89.0)
24-hour UCCR,ug/mmol	>23.00	77.3 (62.2-88.5)	60.7 (51.0-69.8)	43.6 (36.9- 50.6)	87.2 (79.4-92.3)
24-hour UFC, ug	>308.1	57.5 (42.2-71.7)	81.7 (73.5-88.3)	56.3 (44.8-67.0)	82.5 (76.9-86.9)
Predicting cortisol post 1mg DST >5.0 µg/dL					
Spot UCCR, ug/g	>167.36	86.4 (65.1-97.1)	62.1 (53.6-70.0)	25.7 (20.9-31.1)	96.8 (91.2-98.9)
24-hour UCCR,ug/mmol	>29.51	85.0 (62.1-96.8)	77.9 (70.0-84.6)	36.2 (28.2-45.0)	97.2 (92.5-99.0)
24-hour UFC, ug	>333.3	86.4 (65.1-97.1)	81.4 (74.0-87.5)	42.2 (33.2-51.8)	97.4 (93.0-99.1)

The Youden index was used to identify the optimal diagnostic threshold(cut-off value) for cortisol measurements. Sensitivity, specificity, positive predictive value (PPV), and negative predictive value (NPV) were calculated at the optimal cut-off value, and their 95% confidence intervals (CIs) were estimated using the exact binomial method.

DST, dexamethasone suppression test; UCCR, urinary cortisol-to-creatinine ratio; UFC, urinary free cortisol.

### 3.4 Diagnostic accuracy of urinary cortisol tests across subgroups stratified by diabetes status, sex, age, and renal function

**Figure 4:**
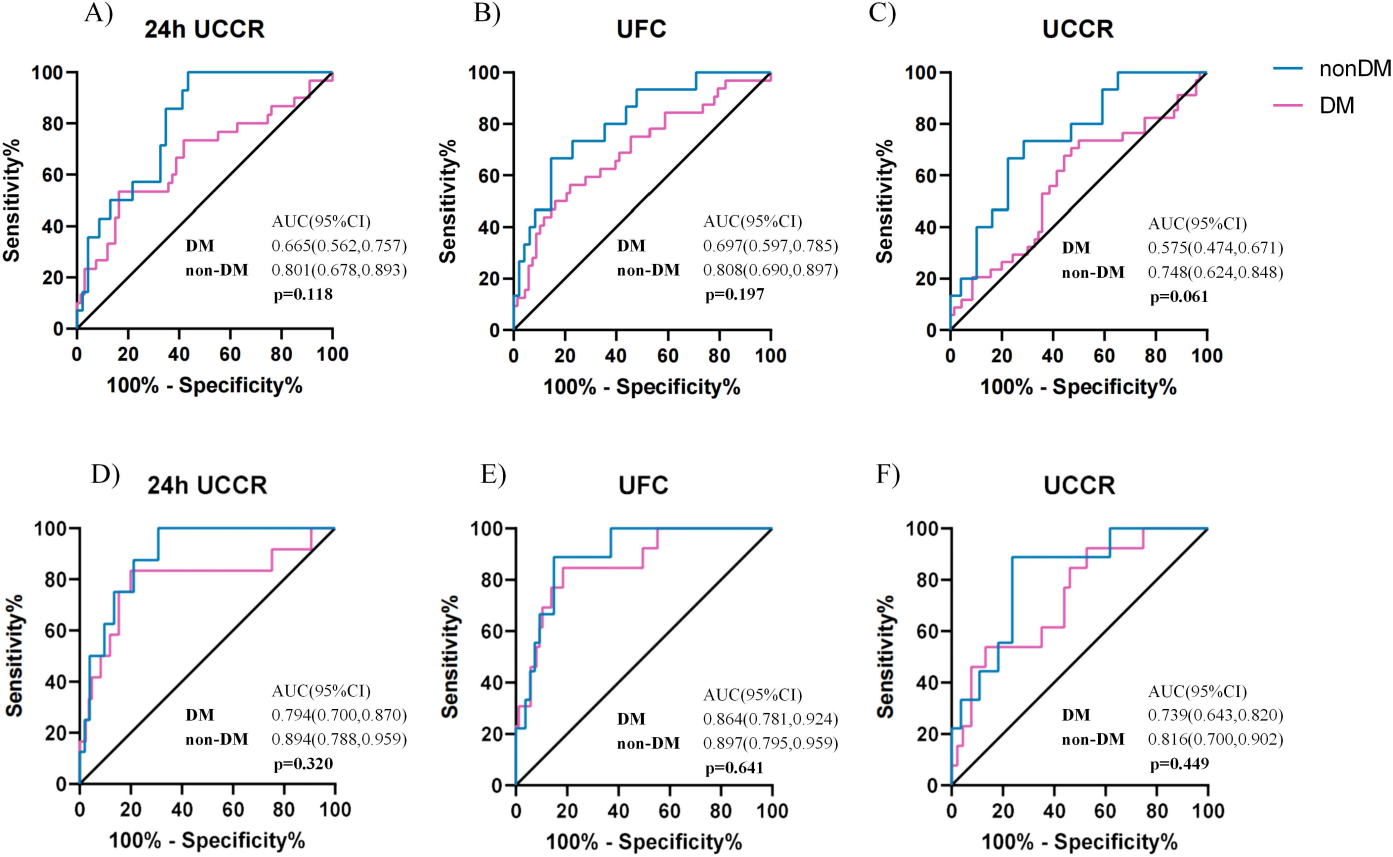
Comparison of diagnostic performance of urinary cortisol tests in patients with or without diabetes. ROC curves for spot UCCR, 24-hour UCCR, and 24-hour UFC in predicting cortisol post DST >1.8 µg/dL, stratified by diabetes status **(A-C)**. ROC curves for predicting cortisol post-1mg DST >5 µg/dL **(D-F)**. Area under the curve (AUC) with exact binomial 95% confidence intervals (95% CI) were calculated. Statistical significance (p) for AUC comparisons between diabetes (n=103) and non-diabetes (n=64) subgroups was assessed using the Delong test. Solid lines indicate the reference (AUC = 0.5, no discrimination). DST-dexamethasone suppression test; UCCR-urinary cortisol-to-creatinine ratio; UFC-urinary free cortisol.

To evaluate the diagnostic accuracy of urinary cortisol measures, participants were stratified by diabetes status (DM vs. non-DM). No statistically significant differences in AUCs were found between the DM and non-DM groups across the various urinary cortisol indices at both the 1.8 µg/dL and 5.0 µg/dL DST thresholds (all p > 0.05, **Figure 4**). However, based on the 1mg DST threshold of 1.8 µg/dL, the ROC AUC for morning spot UCCR was 0.575 (0.474, 0.671) in the DM group and 0.748 (0.624,0.848) in the non-DM group (p=0.061), suggesting a potential trend toward reduced performance in the DM group. At the higher threshold of 5.0 µg/dL, all urinary cortisol tests showed comparable performance between the DM and non-DM groups, with morning spot UCCR achieving an ROC AUC of 0.739 (95% CI, 0.643-0.820) in the DM group and 0.816 (95% CI, 0.700-0.902) in the non-DM group.

In the DM group, morning spot UCCR demonstrated the highest sensitivity for predicting cortisol >1.8 µg/dL post-1mg DST at 73.5%, with lowest specificity, lowest PPV, and comparable NPV of 50.0%, 41.7%, and 79.5%, respectively. For predicting cortisol >5.0 µg/dL post-1mg DST, the specificity and NPV increased to 86.8% and 92.9%, respectively(**Supplementary Table 2**). In comparison, in the non-DM group, morning spot UCCR exhibited better diagnostic accuracy, with sensitivity, specificity, PPV, and NPV of 88.9%, 76.4%, 38.1%, and 97.7%, respectively (**Supplementary Table 3**).

**Figure 5:**
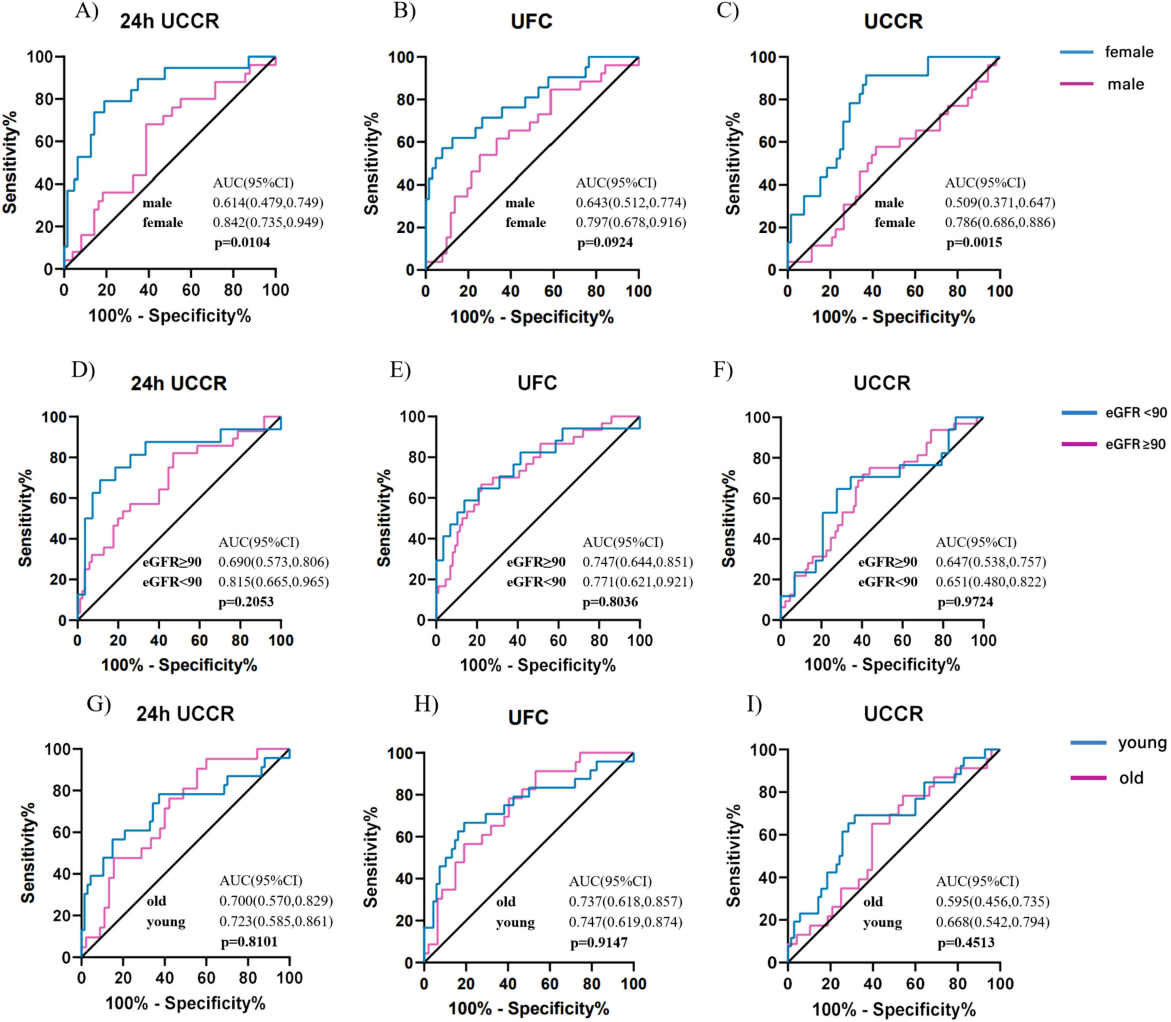
Diagnostic accuracy of urinary cortisol tests across subgroups stratified by sex, age, and renal function for predicting cortisol post DST>1.8 µg/dl. ROC curves for 24-hour UCCR, 24-hour UFC, and morning spot UCCR in predicting cortisol levels post DST >1.8 µg/dL, stratified by sex **(A-C)**, renal function **(D-F)**, and age **(G-I)**. Areas under the curve (AUCs) with exact binomial 95% confidence intervals (95% CI) were calculated for each subgroup, and between-group comparisons were assessed using the Delong test. Solid lines indicate the reference line (AUC = 0.5, no discrimination). DST-dexamethasone suppression test; UCCR-urinary cortisol-to-creatinine ratio; UFC-urinary free cortisol; eGFR-estimated glomerular filtration rate.

To further characterize sex-specific diagnostic performance, subgroup analyses were conducted in men and women. At the 1mg DST threshold of 1.8 µg/dL, females showed higher AUCs than males across all urinary cortisol tests (**Figure 5A-C**), particularly for morning spot UCCR (0.786 vs. 0.509, p = 0.0015). At the higher threshold of 5.0 µg/dL (**Supplementary Figure 2**), the sex difference diminished, with comparable AUCs between males and females (all p > 0.05). In females, morning spot UCCR demonstrated consistently high sensitivity and NPV at both thresholds, whereas in males, the overall diagnostic performance remained moderate(**Supplementary Tables 4**-**5**).

Similarly, stratified analyses by renal function and age revealed no significant differences in diagnostic performance across subgroups (**Figure5 D-I**). NPVs for all urinary cortisol tests remained consistently high across renal and age strata, as summarized in **Supplementary Tables 6**-**9**.

## 4 Discussion

In this hospital-based cross-sectional study, morning spot UCCR emerged as a reliable, non-invasive tool for the initial assessment of cortisol secretion. It correlated well with 24-hour UFC, predicted 1 mg DST results comparably to 24-hour tests, and showed high sensitivity and NPVs for predicting cortisol >1.8 µg/dL and 5.0µg/dL. The diagnostic performance was consistent across subgroups stratified by renal function and age, with a borderline decrease observed in patients with diabetes and a significant decrease in males.

The evaluation and diagnosis of Cushing syndrome remain challenging, particularly in detecting early or mild cortisol excess. This is not only due to the diverse and nonspecific clinical manifestations of Cushing syndrome, but also to the limited accessibility of current diagnostic tools (26). To the best of our knowledge, this is the first study to evaluate the utility of morning spot UCCR in predicting cortisol post 1mg DST results and to compare its effectiveness with that of 24-hour UFC and 24-hour UCCR. Previous studies have reported the use of the urinary free cortisol-to-creatinine ratio from 24-hour and late-night (10:00-11:00 PM) urine collections in the evaluation of Cushing’s syndrome and subclinical Cushing’s syndrome. The 24-hour UCCR has been described as a novel index for distinguishing Cushing’s syndrome from simple obesity and is believed to be superior to 24-hour UFC, according to a recent cross-sectional study (27). The findings of Bruno et al. demonstrated that both 10:00-11:00 PM urinary free cortisol-to-creatinine ratio and late-night 11:00 PM salivary cortisol exhibited similarly high sensitivity and specificity for diagnosing Cushing’s syndrome (28). However, as previously discussed, timed urine collections remain inconvenient and are associated with poor patient compliance, particularly in outpatient settings (29). In fact, our morning spot UCCR reflects overnight urine accumulation—from bedtime to wake-up—a physiologically stable period for most individuals. Requiring only a single morning urine sample, it simplifies the collection process while maintaining comparable diagnostic performance, as demonstrated in our study. Its suitability for outpatient or home-based use further enhances its accessibility and practicality as a routine screening tool for hypercortisolism.

Our study demonstrated that morning spot UCCR maintained a favorable balance of sensitivity and specificity, with excellent NPVs, when evaluated against both DST thresholds. While the 5.0 µg/dL post-DST cortisol threshold has traditionally been applied to screen for overt hypercortisolism, more recent guidelines recommend a lower threshold of 1.8 µg/dL for identifying mild autonomous cortisol secretion (MACS) in patients with adrenal incidentaloma and without overt signs of hypercortisolism (14). Given the low prevalence of participants with positive DST in our cohort, the high NPVs and relatively lower PPVs were expected. Other screening tools, including 24-hour UFC and 24-hour UCCR in our study, as well as LNSC evaluated in previous studies (30), have demonstrated a similar pattern. Mild autonomous cortisol secretion affecting approximately 12% of those with adrenal incidentalomas and up to 22% of patients with primary aldosteronism (6, 7). Our data indicate that when applying the cutoff value of 1.8 µg/dL, currently recommended for defining MACS, the morning spot UCCR yields a NPV of 83.5% for excluding MACS, comparable to that of 24-hour UFC and 24-hour UCCR. These findings support the clinical utility of morning spot UCCR as a convenient outpatient screening tool for ruling out both overt cortisol excess and mild autonomous cortisol secretion. In clinical practice, for patients with resistant hypertension or adrenal incidentaloma without overt features of Cushing’s syndrome, a morning spot UCCR can be used as a rapid screening test. If the result is negative, further evaluations with the 1-mg DST and 24-hour UFC may potentially be avoided.

Excess cortisol contributes to metabolic dysfunction and disrupts glucose homeostasis, leading to insulin resistance and subsequently, diabetes (31). Patients with hypercortisolism generally experience improved glycemic control in diabetes following the normalization of cortisol levels, underscoring the critical role of identifying hypercortisolism in improving clinical outcome (32, 33). To date, the best test for screening hypercortisolism in patients with diabetes is still in dispute (34, 35). A recent review highlighted that the available data are not sufficiently robust to support widespread and indiscriminate screening for endogenous hypercortisolism in patients with type 2 diabetes or obesity due to cost-effectiveness concerns (36). A previous study by Steffensen et al., which used the DST>50 nmol/L(1.8µg/dL) as the gold standard in patients with diabetes, found that LNSC had a very low specificity of 14%, with an area under the ROC curve of 0.57 (37). Using the same cut-off value, our data showed that morning spot UCCR achieved a comparable overall accuracy of 0.58 in patients with diabetes, with a more balanced sensitivity and specificity (73.5% and 50.0%, respectively). Although no statistically significant difference was observed compared with patients without diabetes, a slight downward trend in AUC was noted, suggesting that the diagnostic performance of morning spot UCCR may be slightly reduced in patients with diabetes. This discrepancy may be explained by the background hyperactivity of the HPA axis in patients with diabetes and thus may reduce the distinction between normal and pathological cortisol states, thereby diminishing the discriminative power of morning spot UCCR in this subgroup. The bidirectional relationship between hypercortisolism and diabetes has been well-documented (38–40). Daily cortisol output is often disrupted in patients with diabetes, particularly in those experiencing diabetes-related distress (41) or chronic complications (42), potentially mediated by dysregulation of the HPA axis secondary to chronic metabolic stress and systemic inflammation (43). This phenomenon is further supported by studies demonstrating that cortisol dysregulation is associated with poor glycemic control and heightened psychological stress (44). Consequently, morning spot UCCR, along with LNSC, 24-hour UFC, and 24-hour UCCR, may not be suitable as a stand-alone screening test for hypercortisolism in this population. Further methodological and clinical investigations are warranted to identify the most reliable biomarker for assessing cortisol status in type 2 diabetes.

In the stratified analyses, the substantially higher AUC of the urinary cortisol tests observed in females suggests a potential sex-related difference in cortisol metabolism. In a large population-based study involving over 4000 participants, Kline et al. also demonstrated a significant sex-related difference and a minor age effect in urinary cortisol excretion, with male patients showing substantially higher 24-hour UFC levels than females (45). A population-based study confirmed that men typically exhibit significantly higher excretion of nearly all urinary steroid metabolites, many of which share close structural similarity with cortisol (46). When quantified using immunoassays, these structurally related steroids may cause cross-reactivity, resulting in overestimation of urinary cortisol concentrations. This physiological difference could partly explain the superior discriminative ability of morning spot UCCR in females. Stratification by renal function showed that most urinary cortisol indices maintained comparable diagnostic accuracy across eGFR categories, suggesting that mild-to-moderate renal impairment did not substantially influence the interpretability of these measures. Age-stratified analyses likewise demonstrated broadly consistent diagnostic performance between younger and older participants.

Given the constraints on healthcare resources in China, inpatient dexamethasone suppression testing is often not feasible, and patient adherence to outpatient DST protocols is generally suboptimal, indicating a pressing need for a more practical alternative for screening cortisol excess. Our study provides initial evidence supporting the utility of morning spot UCCR as a novel biomarker for screening cortisol secretion abnormalities. We conducted all tests within a short time frame to minimize fluctuations in cortisol production. However, certain limitations should be acknowledged. Serum dexamethasone concentrations after DST has not been measured, and therefore the adequacy of dexamethasone suppression could not be confirmed. Currently, mass spectrometry is recognized as the state-of-the-art technique for cortisol measurement. Although previous studies have reported comparable diagnostic performance between immunoassay-based and mass spectrometry-based urinary free cortisol assessments (47, 48), the use of immunoassay as a reference method introduces an inherent limitation. Immunoassays are prone to cross-reactivity with cortisol metabolites and may overestimate cortisol concentrations, particularly in saliva and urine, thereby potentially compromising diagnostic accuracy. In contrast, liquid chromatography–tandem mass spectrometry (LC-MS/MS) offers superior analytical specificity and reproducibility, with markedly reduced inter-laboratory variability. Therefore, future validation of the morning spot UCCR against LC-MS/MS is essential to confirm its diagnostic reliability and to establish it as a robust alternative to the conventional 24-hour UFC measurement. In our study, spot UCCR and 24-hour UCCR were measured by different creatinine assays (Jaffé and enzymatic methods) according to the routine laboratory protocols of our hospital. Generally, enzymatic assays are considered less biased and less susceptible to interference than the Jaffé method standardized to reference material (49). These methods may introduce systematic bias in the ratio. However, the Jaffé method offers a faster turnaround time and therefore remains widely used in clinical practice. Besides, as the morning spot urine sample was collected from the first void after waking, we were unable to assess how factors such as poor sleep quality or nocturia might affect the viability of morning spot UCCR. Moreover, as all participants in our study were hospitalized patients, with a high proportion of adrenal incidentalomas or metabolic comorbidities, this highly selected population may limit the generalizability of our findings to outpatient or community-based screening settings. Furthermore, hospitalized patients with complicated or decompensated diabetes are not equivalent to those typically seen in outpatient settings. A previous study reported only 3.72% of 993 patients attending diabetes outpatient clinic had cortisol levels ≥1.8 µg/dL post-1mg DST (50), which is substantially lower than that observed in our cohort. The fact that our analysis focused on a Chinese population might also hamper the extensibility to the general population. Therefore, validation in independent outpatient cohorts is necessary to confirm the generalizability and reliability of morning spot UCCR.

In conclusion, this hospital-based study indicated that morning spot UCCR serves as a promising alternative to traditional methods for the initial evaluation of cortisol secretion. Its high negative predictive value, combined with its simplicity and non-invasive nature, makes morning spot UCCR a practical option for routine screening. The diagnostic accuracy was consistent across patients stratified by age and renal function, and was slightly affected by sex and diabetes status. Further studies are warranted to validate the clinical utility of morning spot UCCR and to optimize screening protocols for ruling out mild autonomous cortisol secretion.

## Data Availability

The raw data supporting the conclusions of this article will be made available by the authors, without undue reservation.
