# Supplementary material for: Morning spot urinary cortisol-to-creatinine ratio: a novel screening tool for assessing excess cortisol secretion
Source: Front Endocrinol (Lausanne). 2025 Nov 19;16:1663619. doi: 10.3389/fendo.2025.1663619 (PMC12672292; doi:10.3389/fendo.2025.1663619)
Supplement: Supplementary file 1 [file DataSheet1.docx]

Supplementary Figure 1. Variable selection using least absolute shrinkage and selection operator (LASSO) logistic regression.

Twenty-five variables potentially associated with the 1mg DST outcome were included in the LASSO regression analysis. These variables included sex, age, diabetes status, height, weight, BMI, systolic blood pressure, diastolic blood pressure, alanine aminotransferase, aspartate aminotransferase, low-density lipoprotein cholesterol, total cholesterol, triglycerides, high-density lipoprotein cholesterol, estimated glomerular filtration rate, serum creatinine, fasting glucose, sodium, potassium, chloride, fasting C-peptide, 2-hour C-peptide, glycated albumin, glycated hemoglobin A1c, and insulin-like growth factor-1. The optimal penalty term (α = 0.739) was selected using tenfold cross-validation and minimization criterion.

The dotted vertical line was plotted at α = 0.739.


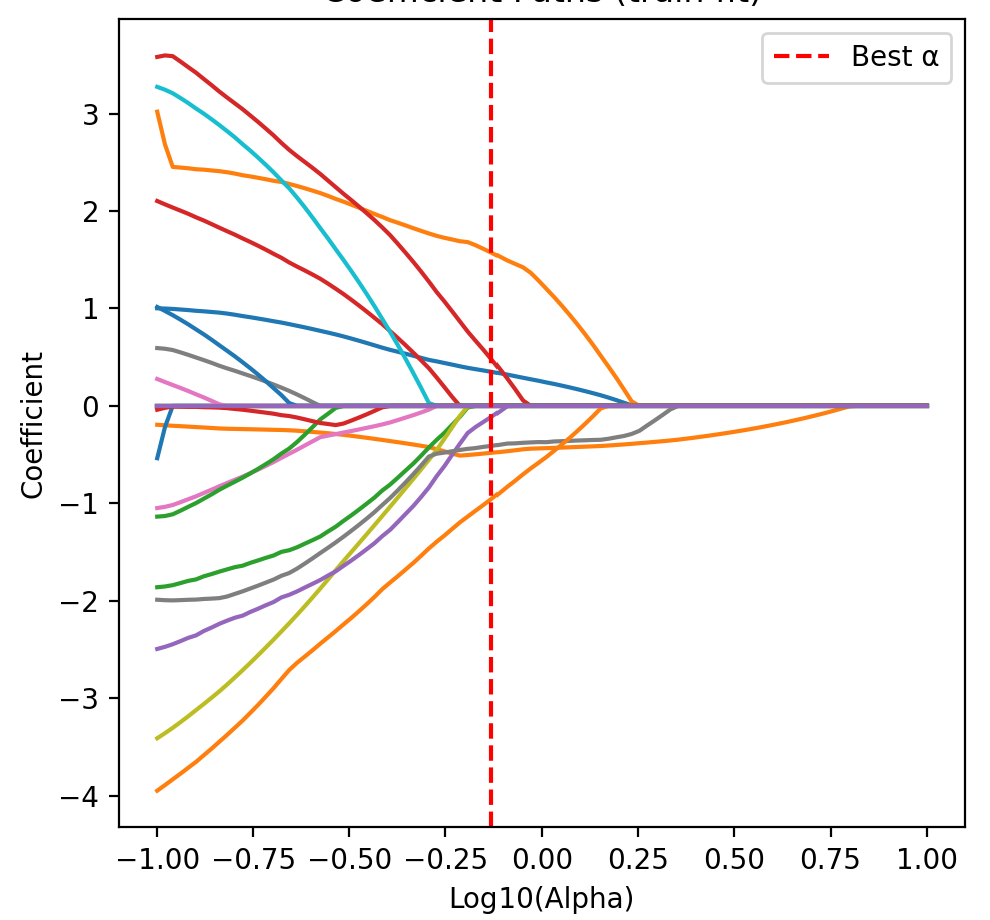


Supplementary Figure 2. Diagnostic accuracy of urinary cortisol tests across subgroups stratified by sex, age, and renal function for cortisol level post DST>5.0ug/dl.

ROC curves for 24-hour UCCR, 24-hour UFC, and morning spot UCCR in predicting cortisol levels post DST >5.0 µg/dL, stratified by sex(A-C), renal function(D-F), and age(G-I). Areas under the curve (AUCs) with exact binomial 95% confidence intervals (95% CI) were calculated for each subgroup, and between-group comparisons were assessed using the Delong test. Solid lines indicate the reference line (AUC = 0.5, no discrimination).

DST-dexamethasone suppression test; UCCR-urinary cortisol-to-creatinine ratio; UFC-urinary free cortisol; eGFR-estimated glomerular filtration rate.


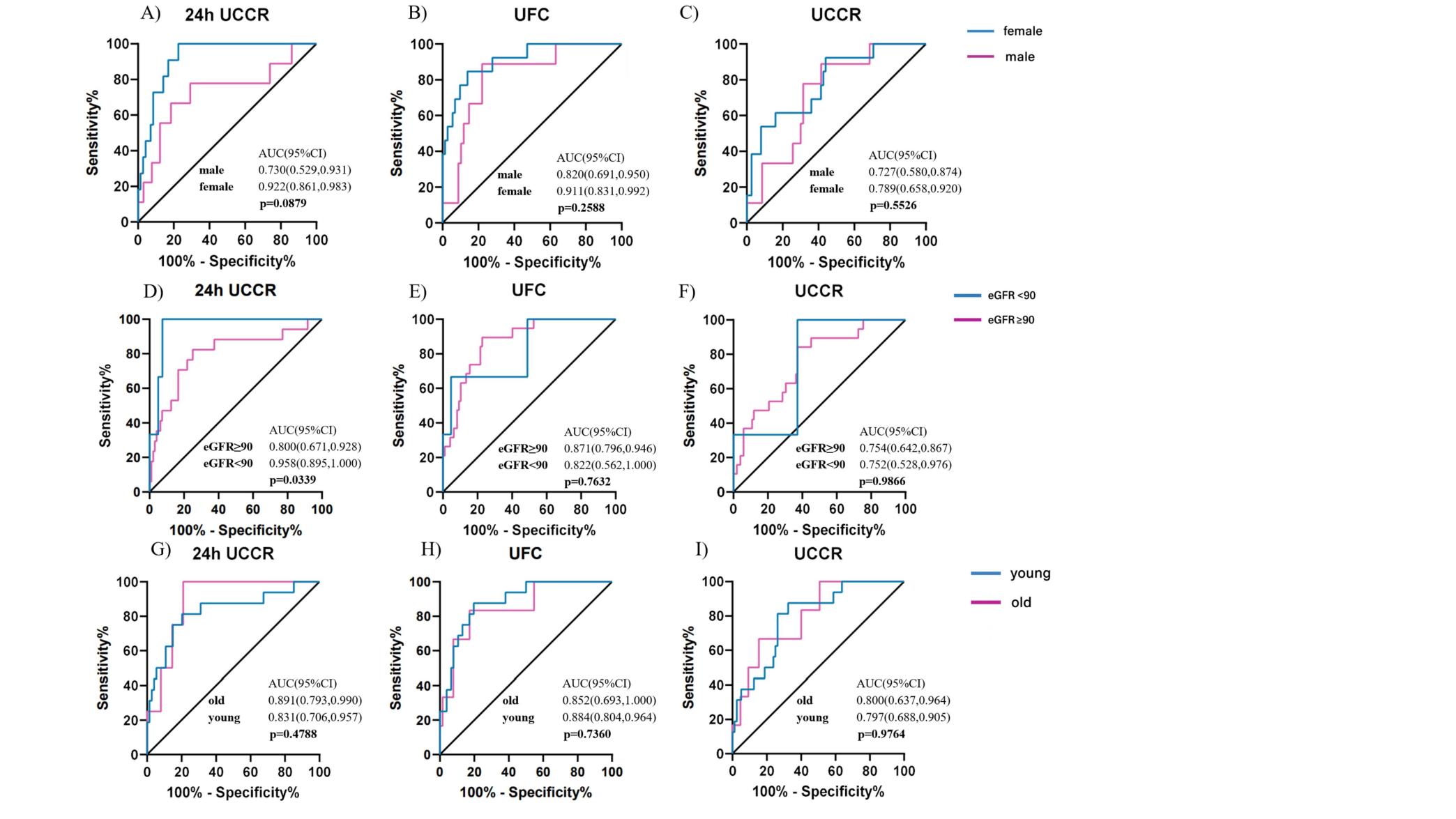


Supplementary Table 1. Variables predicting the 1mg DST result selected by LASSO-logistic regression model.

| **Variables** | **Coefficient** |
| --- | --- |
| Total Cholesterol | 1.5739 |
| 2-hour C-peptide | −0.9578 |
| Diabetes Status | 0.3498 |
| HDL Cholesterol | 0.4822 |
| Sex | −0.4818 |
| Sodium | −0.4112 |
| Estimated GFR | −0.1146 |

Supplementary Table 2. Comparison of spot UCCR, 24h UCCR and 24h UFC at different 1mg DST cutoff values in DM group(n=103).

|  | **Cut-off value** | **Sensitivity (95% CI) (%)** | **Specificity (95% CI) (%)** | **PPV (95% CI) (%)** | **NPV (95% CI) (%)** |
| --- | --- | --- | --- | --- | --- |
| predicting cortisol post 1mg DST >1.8 µg/dL | | | | | |
| Spot UCCR, ug/g | >141.46 | 73.5 (55.6-87.1) | 50.0 (37.8-62.2) | 41.7 (34.4-49.3) | 79.5 (67.9-87.7) |
| 24h UCCR, ug/mmol | >30.41 | 53.3 (34.3-71.7) | 83.6 (72.5-91.5) | 59.3 (43.5-73.3) | 80.0 (72.9-85.6) |
| 24-hour UFC, ug | >286.2 | 56.3 (37.7-73.6) | 77.9 (66.2-87.1) | 54.5 (41.1-67.3) | 79.1 (71.5-85.1) |
| predicting cortisol post 1mg DST >5.0 µg/dL | | | | | |
| Spot UCCR, ug/g | >316.92 | 53.9 (25.1-80.8) | 86.8 (78.1-93.0) | 36.8 (22.0-54.7) | 92.9 (87.9-96.0) |
| 24h UCCR, ug/mmol | >30.41 | 83.3 (51.6-97.9) | 80.0 (69.9-87.9) | 37.0 (26.4-49.1) | 97.1 (90.5-99.2) |
| 24-hour UFC, ug | >336.7 | 84.6 (54.6-98.1)) | 81.6 (71.9-89.1) | 40.7 (29.4-53.1) | 97.3 (90.8-99.2) |

Note: The Youden index was used to identify the optimal diagnostic threshold(cut-off value) for cortisol measurements. Sensitivity, specificity, positive predictive value (PPV), and negative predictive value (NPV) were calculated at the optimal cut-off value, and their 95% confidence intervals (CIs) were estimated using the exact binomial method.

Abbreviations: DST, dexamethasone suppression test; UCCR, urinary cortisol-to-creatinine ratio; UFC, urinary free cortisol.

Supplementary Table 3. Comparison of spot UCCR, 24h UCCR and 24h UFC at different 1mg DST cutoff values in non-DM group(n=64).

|  | **Cut-off value** | **Sensitivity (95% CI) (%)** | **Specificity (95% CI) (%)** | **PPV (95% CI) (%)** | **NPV (95% CI) (%)** |
| --- | --- | --- | --- | --- | --- |
| predicting cortisol post 1mg DST >1.8 µg/dL | | | | | |
| Spot UCCR, ug/g | >149.04 | 73.3 (44.9-92.2) | 71.4 (56.7-83.4) | 44.0 (31.5-57.4) | 89.7 (78.8-95.4) |
| 24h UCCR,ug/mmol | >19.32 | 100.0 (76.8-100.0) | 56.5 (41.1-71.1) | 41.2 (33.5-49.3) | 100.0 (-) |
| 24-hour UFC, ug | >306.7 | 66.7 (38.4-88.2) | 85.4 (72.2-93.9) | 58.8 (39.8-75.6) | 89.1 (79.9-94.4) |
| predicting cortisol post 1mg DST >5.0 µg/dL | | | | | |
| Spot UCCR, ug/g | >167.36 | 88.9 (51.8-99.7) | 76.4 (63.0-86.8) | 38.1 (26.6-51.1) | 97.7 (86.8-99.6) |
| 24h UCCR,ug/mmol | >26.25 | 100.0 (63.1-100.0) | 69.2 (54.9-81.3) | 33.3 (25.0-42.9) | 100.0 (-) |
| 24-hour UFC, ug | >310.6 | 88.9 (51.8-99.7) | 85.2 (72.9-93.4) | 50.0 (33.6-66.4) | 97.9 (87.8-99.7) |

Note: The Youden index was used to identify the optimal diagnostic threshold(cut-off value) for cortisol measurements. Sensitivity, specificity, positive predictive value (PPV), and negative predictive value (NPV) were calculated at the optimal cut-off value, and their 95% confidence intervals (CIs) were estimated using the exact binomial method.

Abbreviations: DST, dexamethasone suppression test; UCCR, urinary cortisol-to-creatinine ratio; UFC, urinary free cortisol.

Supplementary Table 4. Comparison of spot UCCR, 24h UCCR and 24h UFC at different 1mg DST cutoff values in women(n=88).

|  | **Cut-off value** | **Sensitivity (95% CI) (%)** | **Specificity (95% CI) (%)** | **PPV (95% CI) (%)** | **NPV (95% CI) (%)** |
| --- | --- | --- | --- | --- | --- |
| predicting cortisol post 1mg DST >1.8 µg/dL | | | | | |
| Spot UCCR, ug/g | >167.36 | 91.3 (72.0-98.9) | 63.1 (50.2-74.7) | 46.7 (38.3-55.2) | 95.3 (84.3-98.7) |
| 24h UCCR,ug/mmol | >29.39 | 78.9 (54.4-93.9) | 81.0 (69.1-89.8) | 55.6 (41.7-68.6) | 92.7 (84.1-96.8) |
| 24-hour UFC, ug | >297.20 | 61.9 (38.4-81.9) | 87.5 (76.8-94.4) | 61.9 (43.9-77.1) | 87.5 (80.1-92.4) |
| predicting cortisol post 1mg DST >5.0 µg/dL | | | | | |
| Spot UCCR, ug/g | >167.36 | 92.3 (64.0-99.8) | 56.0 (44.1-67.5) | 26.7 (21.2-32.9)) | 97.7 (86.3-99.6) |
| 24h UCCR,ug/mmol | >29.39 | 100.0 (71.5-100.0) | 77.5 (66.0-86.5 | 40.7 (30.9-51.4) | 100.0 (—) |
| 24-hour UFC, ug | >297.20 | 84.6 (54.6-98.1) | 86.1 (75.9-93.1) | 52.4 (37.2-67.2) | 96.9 (89.6-99.1) |

Note: The Youden index was used to identify the optimal diagnostic threshold(cut-off value) for cortisol measurements. Sensitivity, specificity, positive predictive value (PPV), and negative predictive value (NPV) were calculated at the optimal cut-off value, and their 95% confidence intervals (CIs) were estimated using the exact binomial method.

Abbreviations: DST, dexamethasone suppression test; UCCR, urinary cortisol-to-creatinine ratio; UFC, urinary free cortisol.

Supplementary Table 5. Comparison of spot UCCR, 24h UCCR and 24h UFC at different 1mg DST cutoff values in men(n=79).

|  | **Cut-off value** | **Sensitivity (95% CI) (%)** | **Specificity (95% CI) (%)** | **PPV (95% CI) (%)** | **NPV (95% CI) (%)** |
| --- | --- | --- | --- | --- | --- |
| predicting cortisol post 1mg DST >1.8 µg/dL | | | | | |
| Spot UCCR, ug/g | >142,71 | 57.7 (36.9-76.6) | 58.5 (44.1-71.9) | 40.5 (30.1-51.9) | 73.8 (63.0-82.3) |
| 24h UCCR,ug/mmol | >22.99 | 68.0 (46.5-85.1) | 61.2 (46.2-74.8) | 47.2 (36.5-58.2) | 78.9 (67.0-87.4) |
| 24-hour UFC, ug | >308.10 | 53.9 (33.4-73.4) | 74.5 (60.4-85.7) | 51.9 (37.4-66.0) | 76.0 (67.0-83.2) |
| predicting cortisol post 1mg DST >5.0 µg/dL | | | | | |
| Spot UCCR, ug/g | >142.71 | 88.9 (51.8-99.7) | 58.6 (46.2-70.2) | 21.6 (16.1-28.4) | 97.6 (86.5-99.6) |
| 24h UCCR,ug/mmol | >26.25 | 77.8 (40.0-97.2) | 70.8 (58.2-81.4) | 26.9 (18.0-38.1) | 95.8 (87.0-98.7) |
| 24-hour UFC, ug | >336.70 | 88.9 (51.8-99.7) | 77.9 (66.2-87.1) | 34.8 (24.4-46.9) | 98.1 (89.3-99.7) |

Note: The Youden index was used to identify the optimal diagnostic threshold(cut-off value) for cortisol measurements. Sensitivity, specificity, positive predictive value (PPV), and negative predictive value (NPV) were calculated at the optimal cut-off value, and their 95% confidence intervals (CIs) were estimated using the exact binomial method.

Abbreviations: DST, dexamethasone suppression test; UCCR, urinary cortisol-to-creatinine ratio; UFC, urinary free cortisol.

Supplementary Table 6. Comparison of spot UCCR, 24h UCCR and 24h UFC at different 1mg DST cutoff values in the eGFR < 90 mL/min/1.73 m²group(n=46).

|  | **Cut-off value** | **Sensitivity (95% CI) (%)** | **Specificity (95% CI) (%)** | **PPV (95% CI) (%)** | **NPV (95% CI) (%)** |
| --- | --- | --- | --- | --- | --- |
| predicting cortisol post 1mg DST >1.8 µg/dL | | | | | |
| Spot UCCR, ug/g | >176.45 | 64.7 (38.3-85.8) | 72.4 (52.8-87.3) | 57.9 (40.9-73.2) | 77.8 (63.9-87.4) |
| 24h UCCR,ug/mmol | >23.48 | 68.8 (41.3-89.0) | 88.9 (70.8-97.6) | 78.6 (54.5-91.8) | 82.8 (69.6-90.9) |
| 24-hour UFC, ug | >265.80 | 58.8 (32.9-81.6) | 86.2 (68.3-96.1) | 71.4 (48.1-87.1) | 78.1 (66.5-86.5) |
| predicting cortisol post 1mg DST >5.0 µg/dL | | | | | |
| Spot UCCR, ug/g | >176.45 | 100.0 (29.2-100.0) | 62.8 (46.7-77.0) | 15.8 (11.3-21.7) | 100.0 (-) |
| 24h UCCR,ug/mmol | >35.75 | 100.0 (29.2-100.0) | 92.5 (79.6-98.4) | 50.0 (25.2-74.8) | 100.0 (-) |
| 24-hour UFC, ug | >447.30 | 66.7 (9.4-99.2) | 95.4 (84.2-99.4) | 50.0 (17.2-82.8) | 97.6 (89.2-99.5) |

Note: The Youden index was used to identify the optimal diagnostic threshold(cut-off value) for cortisol measurements. Sensitivity, specificity, positive predictive value (PPV), and negative predictive value (NPV) were calculated at the optimal cut-off value, and their 95% confidence intervals (CIs) were estimated using the exact binomial method.

Abbreviations: DST, dexamethasone suppression test; UCCR, urinary cortisol-to-creatinine ratio; UFC, urinary free cortisol.

Supplementary Table 7. Comparison of spot UCCR, 24h UCCR and 24h UFC at different 1mg DST cutoff values in the eGFR≥90 mL/min/1.73 m²group(n=121).

|  | **Cut-off value** | **Sensitivity (95% CI) (%)** | **Specificity (95% CI) (%)** | **PPV (95% CI) (%)** | **NPV (95% CI) (%)** |
| --- | --- | --- | --- | --- | --- |
| predicting cortisol post 1mg DST >1.8 µg/dL | | | | | |
| Spot UCCR, ug/g | >149.04 | 71.9 (53.3-86.3) | 59.6 (48.6-69.8) | 39.0 (31.4-47.1) | 85.5 (76.7-91.3) |
| 24h UCCR,ug/mmol | >22.99 | 82.1 (63.1-93.9) | 52.9 (41.8-63.9) | 36.5 (30.2-43.3) | 90.0 (79.9-95.3) |
| 24-hour UFC, ug | >333.30 | 66.7 (47.2-82.7) | 77.9 (67.7-86.1) | 51.3 (39.7-62.8) | 87.0 (80.0-91.8) |
| predicting cortisol post 1mg DST >5.0 µg/dL | | | | | |
| Spot UCCR, ug/g | >167.36 | 84.2 (60.4-96.6) | 62.8 (52.6-72.1) | 29.6 (23.4-36.7) | 95.5 (88.2-98.4) |
| 24h UCCR,ug/mmol | >29.51 | 82.4 (56.6-96.2) | 75.0 (65.1-83.3) | 36.8 (27.9-46.8) | 96.0 (89.5-98.5) |
| 24-hour UFC, ug | >333.30 | 89.5 (66.9-98.7) | 77.3 (67.7-85.2) | 43.6 (34.2-53.5) | 97.4 (91.0-99.3) |

Note: The Youden index was used to identify the optimal diagnostic threshold(cut-off value) for cortisol measurements. Sensitivity, specificity, positive predictive value (PPV), and negative predictive value (NPV) were calculated at the optimal cut-off value, and their 95% confidence intervals (CIs) were estimated using the exact binomial method.

Abbreviations: DST, dexamethasone suppression test; UCCR, urinary cortisol-to-creatinine ratio; UFC, urinary free cortisol.

Supplementary Table 8. Comparison of spot UCCR, 24h UCCR and 24h UFC at different 1mg DST cutoff values in the age≥60 years group (n=71).

|  | **Cut-off value** | **Sensitivity (95% CI) (%)** | **Specificity (95% CI) (%)** | **PPV (95% CI) (%)** | **NPV (95% CI) (%)** |
| --- | --- | --- | --- | --- | --- |
| predicting cortisol post 1mg DST >1.8 µg/dL | | | | | |
| Spot UCCR, ug/g | >194.05 | 65.2 (42.7-83.6) | 60.4 (45.3-74.2) | 44.1 (33.3-55.6) | 78.4 (66.4-86.9) |
| 24h UCCR,ug/mmol | >18.76 | 95.2 (76.2-99.9) | 40.0 (25.7-55.7) | 42.6 (36.4-48.9) | 94.7 (72.0-99.2) |
| 24-hour UFC, ug | >185.50 | 91.3 (72.0-98.9) | 46.8 (32.1-61.9) | 45.7 (38.4-53.0) | 91.7 (73.9-97.7) |
| predicting cortisol post 1mg DST >5.0 µg/dL | | | | | |
| Spot UCCR, ug/g | >304.76 | 66.7 (22.3-95.7) | 84.6 (73.5-92.4) | 28.6 (15.2-47.2) | 96.5 (89.8-98.8) |
| 24h UCCR,ug/mmol | >30.41 | 100.0 (39.8-100.0) | 79.0 (66.8-88.3) | 23.5 (16.0-33.3) | 100.0 (-) |
| 24-hour UFC, ug | >327.60 | 83.3 (35.9-99.6) | 82.8 (71.3-91.1) | 31.2 (19.2-46.4) | 98.1 (89.8-99.7) |

Note: The Youden index was used to identify the optimal diagnostic threshold(cut-off value) for cortisol measurements. Sensitivity, specificity, positive predictive value (PPV), and negative predictive value (NPV) were calculated at the optimal cut-off value, and their 95% confidence intervals (CIs) were estimated using the exact binomial method.

Abbreviations: DST, dexamethasone suppression test; UCCR, urinary cortisol-to-creatinine ratio; UFC, urinary free cortisol.

Supplementary Table 9. Comparison of spot UCCR, 24h UCCR and 24h UFC at different 1mg DST cutoff values in the age<60 years group(n=96).

|  | **Cut-off value** | **Sensitivity (95% CI) (%)** | **Specificity (95% CI) (%)** | **PPV (95% CI) (%)** | **NPV (95% CI) (%)** |
| --- | --- | --- | --- | --- | --- |
| predicting cortisol post 1mg DST >1.8 µg/dL | | | | | |
| Spot UCCR, ug/g | >142.71 | 69.2 (48.2-85.7) | 68.6 (56.4-79.1) | 45.0 (34.7-55.7) | 85.7 (76.7-91.6) |
| 24h UCCR,ug/mmol | >31.25 | 56.5 (34.5-76.8) | 85.1 (74.3-92.6) | 56.5 (39.8-71.9) | 85.1 (78.0-90.2) |
| 24-hour UFC, ug | >333.30 | 66.7 (44.7-84.4) | 80.9 (69.5-89.4) | 55.2 (41.2-68.4) | 87.3 (79.4-92.5) |
| predicting cortisol post 1mg DST >5.0 µg/dL | | | | | |
| Spot UCCR, ug/g | >142.71 | 87.5 (61.7-98.4) | 67.5 (56.1-77.6) | 35.0 (27.2-43.7) | 96.4 (88.0-99.0) |
| 24h UCCR,ug/mmol | >29.18 | 81.3 (54.4-96.0) | 79.7 (68.8-88.2) | 46.4 (34.2-59.1) | 95.2 (87.6-98.2) |
| 24-hour UFC, ug | >333.30 | 87.5 (61.7-98.4) | 80.3 (69.5-88.5) | 48.3 (36.4-60.4) | 96.8 (89.2-99.1) |

Note: The Youden index was used to identify the optimal diagnostic threshold(cut-off value) for cortisol measurements. Sensitivity, specificity, positive predictive value (PPV), and negative predictive value (NPV) were calculated at the optimal cut-off value, and their 95% confidence intervals (CIs) were estimated using the exact binomial method.

Abbreviations: DST, dexamethasone suppression test; UCCR, urinary cortisol-to-creatinine ratio; UFC, urinary free cortisol.
